# Clinical, Genetic, and Immunological Spectrum of CHAI and LATAIE Patients from a Tertiary Referral Centre in India

**DOI:** 10.3390/ijms27010014

**Published:** 2025-12-19

**Authors:** Priyanka Setia, Umair Ahmed Bargir, Mukesh Desai, Aparna Dalvi, Shweta Shinde, Neha Jodhawat, Pallavi Gaikwad, Sagar Bhattad, Chandrakala Shainmukhaih, Maya Gupta, Amruta Dhawale, Priyanka Kambli, Reetika Malik Yadav, Manas Kalra, Harikrishnan Gangadharan, Meena Sivasankaran, Vibha Bafna, Prawin Kumar, Priya Sarvanan, Mamta Manglani, Ratna Sharma, Parag Tamhankar, Manisha Madkaikar

**Affiliations:** 1 Indian Council of Medical Research -National Institute of Immunohematology (ICMR -NIIH), 13th Floor, New Multistorey Building, KEM Hospital, Parel, Mumbai 400012, India; priyankasetia19@gmail.com (P.S.); drumairbargir@gmail.com (U.A.B.); aparna.dalvi@yahoo.co.in (A.D.); shwetashinde0911@gmail.com (S.S.); nehajodhawat7@gmail.com (N.J.); g.pallavig12@gmail.com (P.G.); maya_rk@rediffmail.com (M.G.); amrutadhawale731@gmail.com (A.D.); priyanka.kambli@gmail.com (P.K.); reetikamalik@gmail.com (R.M.Y.); 2 Department of Immunology, Bai Jerbai Wadia Children’s Hospital, Parel, Mumbai 400012, India; mmdesai007@gmail.com; 3 Department of Paediatrics, Aster CMI Hospital, Bangalore 560092, India; drsagarbhattad@gmail.com; 4 Department of Haematology, 10th Floor, New Multistorey Building, KEM Hospital, Parel, Mumbai 400012, India; drchandra_s@rediffmail.com; 5 Department of Pediatric Haematology and Oncology, Sir Ganga Ram Hospital, New Delhi 110060, India; manaskalra27@gmail.com; 6 Department of Rheumatology, Government Medical College, Kottayam 686008, India; drharikrishnang@gmail.com; 7 Department of Pediatric Hemato-Oncology and BMT, Kanchi Kamakoti CHILDS Trust Hospital, Chennai 600034, India; meenasankaran04@gmail.com; 8 Department of Pediatric Haematology, Bhartiya Vidyapeeth, Pune 411045, India; vibha_bafna@yahoo.co.in; 9 Department of Paediatrics, AIIMS Jodhpur, Jodhpur 342005, India; drprawin484@gmail.com; 10 Department of Paediatrics, Government Medical College, Kozhikode 673008, India; priya130cmc@gmail.com; 11 Brihanmumbai Municipal Corporation (BMC) CTC, PHO & BMT Centre, Borivali, Mumbai 400066, India; mmanglani@hotmail.com (M.M.); ratnasharma27@gmail.com (R.S.); 12 Centre for Medical Genetics, Mulund, Mumbai 400080, India; paragtmd4@gmail.com

**Keywords:** inborn errors of immunity, PIRD, *LRBA*, *CTLA4*, autoimmunity, immunodeficiency, flow cytometry

## Abstract

Primary immune regulatory disorders (PIRDs) are a group of conditions characterised by a loss of immune tolerance. Two such disorders, CHAI and LATAIE, share common molecular mechanisms, leading to significant clinical overlap. Here, we report demographic, clinical, immunological, and molecular findings in 29 patients referred from different parts of India with a diagnosis of CHAI or LATAIE. LATAIE patients demonstrated a higher prevalence of consanguinity, while CHAI patients more often had a positive family history. Both disorders presented with overlapping clinical features, predominately autoimmune cytopenias, benign lymphoproliferation, and inflammatory bowel disease (IBD). However, the incidence of recurrent infections, otitis media, bronchiectasis, and hypogammaglobulinemia was higher among LATAIE patients as compared to CHAI. Flow cytometry analysis revealed significant differences in T cell subsets, particularly in percentages of CD4+ naïve cells and T regulatory cells (Treg), between the two disorders. B cell abnormalities were also observed. Molecular diagnosis was achieved using targeted or clinical exome sequencing, and specific protein expression was employed to validate the novel variants.

## 1. Introduction

Primary immune regulatory disorders (PIRDs) represent an expanding group within the spectrum of inborn errors of immunity (IEI), characterised by a prominent display of immune-mediated manifestations, including autoimmunity, autoinflammation/hyperinflammation, lymphoproliferation, and malignancy [1]. These disorders primarily arise from defects in the T cell tolerance mechanisms, which play a key role in maintaining immune homeostasis [2].

Advances in genome sequencing have significantly enhanced our understanding of PIRDs by identifying pathogenic mutations in key regulatory molecules essential for maintaining immune balance. Among these, mutations in Cytotoxic T lymphocyte antigen-4 (*CTLA4*) and Lipopolysaccharide-responsive beige-like anchor protein (*LRBA*) have emerged as prominent examples. These monogenic disorders emphasise the crucial role of immune checkpoints in regulating immune responses and emphasise the importance of investigating these pathways in context of PIRDs [3].

Human *CTLA4* mutations were first described in 2014 by two independent research groups [4,5], with a phenotype closely resembling *Ctla4*-knockout mice that develop multiorgan lymphocytic infiltration. These mutations result in a condition termed “*CTLA4* haploinsufficiency with autoimmune infiltration” (CHAI) [6]. *CTLA4* is encoded by four exons on chromosome 2, where exon 1 encodes the leader peptide, exon 2 the extracellular ligand binding domain, exon 3 the transmembrane region, and exon 4 the cytoplasmic tail, and each domain is critical to structure and function of the protein [7,8].

*CTLA4*, also known as CD152, is constitutively expressed on Foxp3+ regulatory T (Tregs) cells, playing a vital role in their homeostasis and suppressive activity [9]. It functions as a negative regulator of T cell activation and is structurally similar to CD28 and ICOS. *CTLA4* binds with higher affinity to costimulatory molecules CD80 and CD86 on antigen-presenting cells (APCs) than CD28. Through trans-endocytosis, *CTLA4* internalises these ligands from the APC surface, thereby inhibiting the “second signal” required for T cell activation through the T cell receptor (TCR) pathway [10,11].

Biallelic mutations in *LRBA* also impair *CTLA4* expression and function, leading to a phenotypically similar but more penetrant disease [12,13,14]. *LRBA* regulates *CTLA4* by interacting with its cytoplasmic tail and preventing lysosome degradation. It was first identified in 2012 as a cause of common variable immunodeficiency (CVID) with autoimmunity [15]; however *LRBA* is now recognised as a heterogeneous disorder with a range of clinical features [16]. Mutations in *LRBA* are referred to as “*LRBA* deficiency with autoantibodies, regulatory T (Treg) cell defects, autoimmune infiltration, and enteropathy” (LATAIE) [6].

*LRBA* is a large protein encoded by 58 exons on chromosome 4 and comprises 2863 amino acids (~319 kDa) [17]. One of its well-characterised domains is the BEACH (beige and Chediak–Higashi) domain, located in the C-terminal region. *LRBA* also contains a ConA-like domain, pleckstrin homology (PH) domain, WD40 repeats, and DUF1088 (domain of unknown function 1088), which may indicate specific functions [17].

To date, more than 200 cases with mutations in *CTLA4* or *LRBA* [7] have been reported worldwide. However, data from Indian population remain scarce. The available literature is limited to a few case reports or small case series discussing these disorders in Indian patients [18,19,20,21,22,23,24].

In this study, we provide a comprehensive analysis of the clinical, immunological, and molecular findings from a cohort of Indian patients diagnosed with CHAI and LATAIE. We also aim to identify potential diagnostic markers that could facilitate recognition of *CTLA4* and *LRBA* deficiencies, despite the overlapping clinical spectrum caused by the shared pathway.

## 2. Results

### 2.1. Demographic Characteristics

A total of 29 patients were included in this study: 17 LATAIE (11 males and 6 females) and 12 CHAI patients (8 males and 4 females). This study includes data samples referred to our centre for evaluation. The demographic comparison between CHAI and LATAIE is summarised in Table 1.

### 2.2. Clinical Spectrum

The clinical manifestations observed in this cohort, is summarised in Table 2. Across both groups, autoimmune cytopenias were the most common clinical feature, with similar frequencies in LATAIE (70.59%) and CHAI (75%) patients (*p*-value > 0.999). In LATAIE patients, Evans syndrome was the most prevalent subtype, observed in 50% (6/12), followed by autoimmune haemolytic anaemia (AIHA) at 25% (3/12), immune thrombocytopenia (ITP) at 16.66% (2/12), and neutropenia at 8.3% (1/12). In contrast, CHAI patients most commonly presented with AIHA (44%, 4/9), followed by ITP and neutropenia (each 22%; 2/9) and Evans syndrome (11%; 1/9).

The second most common complication observed was non-malignant lymphoproliferation, presenting as splenomegaly and lymphadenopathy. Splenomegaly was significantly more prevalent in LATAIE (58.82%) compared to CHAI patients (8.33%) (*p*-value 0.0080). However, lymphadenopathy occurred at similar rates in both groups (41.18% in LATAIE and 50% in CHAI, *p*-value 0.7163).

The third clinical complication was sinopulmonary infections, including pneumonia, sinusitis, and bronchiectasis, which were significantly higher in LATAIE patients (58.82%) than in CHAI patients (8.33%) (*p*-value 0.0080).

Both groups showed comparable frequencies of inflammatory bowel disease (IBD) (affecting 47% LATAIE and 58% CHAI patients, *p*-value 0.7104). Other clinical features noted across the groups included diarrhoea, recurrent infections, failure to thrive, fevers, otitis media, polyarthritis, and autoimmune hepatitis.

Some manifestations were exclusive to LATAIE patients in our cohort, including interstitial lung disease (ILD), vasculitis, and type 1 diabetes. In contrast, one CHAI patient presented with a diagnosis of aplastic anaemia.

### 2.3. Immunological Findings

Immunological evaluation was performed in 20 patients, 14 LATAIE and 6 CHAI patients. Data on individual immune assessment are provided in Supplementary Table S1a,b, while key immune abnormalities noted in the cohort are summarised in Table 3.

Lymphopenia was observed in approximately 35.71% of LATAIE patients, compared to 16.67% of CHAI patients (*p*-value 0.6120). No statistically significant differences were noted in absolute counts of T, Th, and Tc cells between the two groups.

However, a significant difference was observed in the naïve Th compartment. Low Th naïve cells were observed in nine (64.29%) LATAIE patients, while none of CHAI patients exhibited this finding (*p*-value 0.0141). Additionally, Tc naïve cells were reduced in two (14.29%) LATAIE patients, with no CHAI patients showing this feature.

Elevated double-negative T cells (DNTs) (>2.5% of T cells) were observed in both disease categories, without significant difference (*p*-value 0.6380). T regulatory cells (Tregs) were lower in 57.14% of LATAIE patients but within normal ranges for all CHAI patients (*p*-value 0.0699).

In the B cell compartment, 64.29% of LATAIE patients had low B cells, compared to 33.33% of CHAI patients (*p*-value 0.3359). Despite this, both groups showed similar frequencies of B cell abnormalities, including reduced B memory cells, low-class switch memory B cells, and elevated CD21 low B cells.

Immunoglobulin levels were available for 14 out of 17 LATAIE patients and 7 out of 12 CHAI patients. Panhypogammaglobulinemia was more frequent in LATAIE (42.86%) than CHAI (14.29%), though this difference was not statistically significant (*p*-value 0.3371). Notably, low IgG levels were significantly more common in CHAI patients (57.14%) compared to LATAIE patients (7.14%) (*p*-value 0.0251).

For specific protein expression, flow cytometry was used to evaluate the expression of *LRBA* protein in two LATAIE patients (L2 and L6) on activated T cells and compare them to unrelated healthy controls. Both patients showed *LRBA* expression levels below 12%, in contrast to ~70% expression observed in healthy controls.

Furthermore, *CTLA4* protein expression was evaluated on T regulatory cells in four CHAI patients (C1, C2, C4, and C6), and results were compared with unrelated healthy controls. The *CTLA4* expression levels in these patients were 14% (C1), 12% (C2), 24% (C4), and 26% (C6).

### 2.4. Mutation Spectrum

In our cohort of 17 LATAIE patients, we identified a diverse set of 19 variants distributed across the *LRBA* gene, with 8 being novel. Amongst these, nearly 79% (15/19) were homozygous, while 21% (4/19) were compound heterozygous. The mutation types included missense mutations (42.10%, 8/19), frameshift mutations leading to premature termination (31.57%; 6/19), and splice site or nonsense mutations (21.05%; 4/19).

*CTLA4* variants were identified in 12 patients from nine families, comprising eight distinct variants, of which three were novel. The majority of these variants were clustered in the ligand binding and dimerization domain. These included missense (8/12), nonsense (2/12), and frameshift (2/12) mutations.

A schematic representation of the mutation location within protein domains is provided in Supplementary Figures S1 and S2, and a detailed summary of all molecular findings is presented in Table 4. 

### 2.5. Treatment Outcomes

Therapeutic approaches varied across patients, as they were referred from different centres and managed symptomatically. Due to the lack of standardised treatment protocols and consistent follow-up, most patients received only symptomatic therapies, with limited access to targeted treatments.

In our series, nine patients were lost to follow-up, five with LATAIE and four with CHAI. Among them, two LATAIE patients succumbed to pneumonia or acute respiratory distress syndrome (ARDS), while one CHAI patient died from an unknown cause. Detailed patient-specific treatments and outcomes are summarised in Table 4.

In the LATAIE cohort, a wide range of therapeutic approaches were employed, often requiring combinations of two or more agents. IVIG, either alone or in combination, was administered to 5 out of 17 patients (L6, L9, L11, L12, L16, and L17). Sirolimus was given to three patients (L10, L11, and L16), while methotrexate was prescribed to three patients (L5, L6, and L17). Corticosteroids were used for L15 and L17, while MMF and methylprednisolone were given to patient L2. Additional therapies like rituximab (L16), insulin (L15), and cotrimoxazole (L17) were also given. One patient (L13) underwent successful hematopoietic stem cell transplantation (HSCT), highlighting the spectrum of therapeutic options explored for LATAIE.

Among CHAI patients, common therapeutic interventions included intravenous immunoglobulin (IVIG) for hypogammaglobulinemia or ITP (C1 and C11) and a combination of sirolimus and corticosteroids (C4 and C10) for immune dysregulation. Abatacept was administered to C2, leading to resolution of symptoms. One patient with provisional diagnosis of aplastic anaemia (C12) initially required multiple platelet transfusions and was later managed with eltrambopag and menabol. C3, diagnosed with Hodgkin’s lymphoma, received chemotherapy, while his sibling (C6) was treated with cotrimoxazole for recurrent infections. Azathioprine was added to the treatment regimen for one patient (C10), with favourable outcomes.

## 3. Discussion

In this study, we conducted a comprehensive analysis of demographic, clinical, immunological, and molecular data from patients with CHAI and LATAIE, two closely related immune dysregulatory disorders. We aimed to compare and evaluate the similarities and differences between these two conditions.

We observed distinct patterns in consanguinity and family history, reflecting their inheritance patterns. The high consanguinity rate (88.24%) in LATAIE aligns with its autosomal recessive inheritance, while positive family history (58.33%) in CHAI is consistent with the dominant inheritance with variable penetrance [7].

In the LATAIE cohort, the median age of symptom onset was 4.5 years, with a median diagnostic delay of approximately 3 years. These findings are consistent with Gámez-Díaz et al., who reported a median age of onset of 4 years [25,33]. This variability can be attributed to the evolving nature of *LRBA* deficiency, which can initially present with mild infections and normal immunological workup, progressing to more severe manifestations later in life [34]. In contrast CHAI patients had a median age of onset of symptoms at 13 years, closely aligning with Schwab et al., who reported onset at 11 years [34]. The phenotypic heterogeneity in CHAI may be influenced by various factors beyond the genetic code, such as epigenetic modifiers, environmental triggers, and microbial factors [35,36]. Also, the lack of clinical and treatment awareness of CHAI and LATAIE in India, coupled with their diverse manifestations, likely contributes to diagnostic delays.

The clinical spectrum of CHAI and LATAIE is diverse and overlapping, ranging from recurrent infections to immune dysregulation [7]. In our cohort, autoimmune cytopenias (AICs) and benign lymphoproliferation were the most common shared characteristics. Overall, AICs were observed in 72.41% (21/29) of the patients, with the majority presenting with either Evans syndrome or autoimmune haemolytic anaemia (AIHA). Additionally, 50% of the patients also showed lymphoproliferation, such as lymphadenopathy and/or splenomegaly. AICs accompanied by lymphoproliferation significantly increase the likelihood of underlying inborn errors of immunity (IEIs). Recent genetic studies on Evans syndrome indicate that approximately 65% of cases carry pathogenic or likely pathogenic variants in genes related to IEIs. Around 58% also presented with lymphoproliferation [37], and 25% of these cases had variants identified in *LRBA* or *CTLA4,* underscoring their roles in disease pathogenesis [37,38].

Sinopulmonary infections were more frequently observed in LATAIE than CHAI (*p* = 0.0080), consistent with findings from Jamee et al. [7]. In contrast to previous studies, our CHAI cohort exhibited a lower incidence of recurrent infections [34].

Inflammatory bowel disease (IBD) was the third most common manifestation, affecting over 58% of LATAIE and 47% CHAI patients. The interplay between *LRBA*, *CTLA4*, and Tregs likely contributes to IBD pathogenesis. *LRBA* regulates *CTLA4* by preventing its degradation and recycling it to the surface of activated T cells and Tregs. *LRBA* deficiency thus also results in reduced *CTLA4* expression, impairing immunoregulation and predisposing patients to autoimmunity [6]. Experimental models further highlight *CTLA4*’s role in the intestinal immune balance, demonstrating its necessity for Treg-mediated regulation and accumulation of Foxp3+ Treg in the lamina propria, critical for maintaining intestinal homeostasis [39].

Flow cytometry has revolutionised the diagnosis of inborn errors of immunity by providing crucial insights into immune system abnormalities. In our study, there were no significant differences in overall T, B, or NK cells between LATAIE and CHAI. However, comprehensive immunophenotyping revealed key findings as suggested by Abraham R et al. [10]. LATAIE patients showed reduced CD4+ naïve T cell percentages and Treg numbers compared to CHAI. This suggests skewing towards a memory phenotype and dysregulated activation [40]. Charbonnier et al. previously reported similar findings in *LRBA* deficiency, including disrupted T follicular helper (Tfh) cell compartment and increased autoantibody production [40]. Unfortunately, we were unable to assess Tfh in our cohort to complement our findings. However, investigating this subset could be an avenue for future studies in our research.

B cell abnormalities, including low B cell counts, reduced memory B cells, impaired class switching, decreased plasma blasts, and expansion of CD21 low B cells, were also observed in our study, consistent with prior research [25,33,41], but were not statistically significant. LATAIE patients had a higher frequency of low B cell percentages, while CD21 low B cells were more prominent in *CTLA4*-mutated patients. Importantly, laboratory parameters did not always correlate with clinical manifestations; for example low naïve Th cell levels were also seen in patients with autoimmunity, sinopulmonary infections, and enteropathy.

To facilitate diagnosis and validate novel variants, we used flow cytometry to assess protein expression. *LRBA* expression was evaluated in two patients with LATAIE (L2 and L6) and *CTLA4* expression was evaluated in four CHAI patients (C1, C2, C4, and C6).

*LRBA* mutations were dispersed across various domains, most frequently between DUF4704 and WD40. We identified eight novel mutations, including splice sites, deletions, insertions, and missense variants. *LRBA* protein expression was analysed in two patients (L2 and L6), with protein expression less than 12%. For others, variant pathogenicity was confirmed using ACMG guidelines.

In the CHAI cohort, *CTLA4* mutations were predominately clustered in exon 2, encoding the ligand dimerization domain—a recognised hotspot [7]. *CTLA4* protein expression ranged from 12% to 26%, compared to 80% in healthy controls. Patient C2 (I116SfsTer4) had 12% *CTLA4* expression and received abatacept (a fusion protein of *CTLA4*-Ig), resulting in full remission. C4 (Met134Arg) had both reduced expression and a threefold-lower mean fluorescence intensity (MFI). This mutation affects the conserved MYPPPY critical for ligand binding. Prior studies on the (p.Y139C) variant additionally demonstrated reduced expression, defective CD80/CD86 binding, and impaired suppressor activity [31], which could explain the lower MFI observed in this patient. Patient C12 (Tyr177IlefsTer10), with a mutation in the transmembrane region, presented with aplastic anaemia, similar to another reported case with a mutation at position 172 who underwent successful transplantation [42]. In contrast, our patient was treated with eltrambopag and menabol and platelet transfusion but later died of an unknown cause.

Treatment strategies were diverse, reflecting complex clinical presentations and multicentric care approaches. Most patients received symptomatic care and immunomodulatory or immunosuppressive therapies prior to genetic testing [43]. Targeted treatments like sirolimus and abatacept show promise for personalised approaches [10], though abatacept was limited to only one CHAI patient due to limited availability. Only one LATAIE patient underwent a successful transplant. While HSCT is a potential curative option in such disorders [43,44,45], the degree to which CHAI and LATAIE patients will benefit remains uncertain [7].

This study presents detailed clinical, immunological, and molecular findings from 29 patients (17 with *LRBA* and 12 with *CTLA4* variants). However, it has a few limitations, relatively small sample size, incomplete immune evaluations for all patients, and limited protein expression analysis, which restricted our ability to assess the functional impact of novel variants. Further studies addressing these gaps could provide deeper insights into the pathophysiology and management of these disorders.

## 4. Materials and Methods

**Ethics Approval:** This study was approved by the institutional ethics committee of the ICMR-National Institute of Immunohaematology (file no’s: NIIH/IEC/02-2014). Written informed consent was obtained from all patients or their parents (for participants under 18 years of age) before participation, following the principles outlined in the Declaration of Helsinki.

**Patients and samples:** We collated data from 29 patients (age range: 9 months–55 years); 17 of these were LATAIE and 12 were CHAI. Clinical information was available for all patients, including demographic data, such as age, sex, consanguinity, and family history, and key clinical manifestations, such as the age of onset, lymphoproliferation (lymphadenopathy and hepatosplenomegaly), autoimmune phenomena (AIHA, ITP, and neutropenia), other autoimmune diseases, enteropathy, infection count, failure to thrive, pneumonia, and diarrhoea. Peripheral blood was collected in EDTA, plain, and heparin vacutainers.

### 4.1. Immunological Evaluation

Investigations primarily included a complete blood count (CBC) conducted on the Sysmex XS-800i 5-part automated haematological analyser. Serum immunoglobulin levels (Ig levels) were estimated using a semi-automated nephelometer (BNProspec, Siemens), as per the manufacturer’s protocol.

### 4.2. Flow Cytometry (FCM)

Flow cytometry (FCM) was utilised for the enumeration of lymphocyte subsets, B cell immunophenotyping, Treg evaluation, and the analysis of *LRBA* and *CTLA4* protein expression. Antibodies for these experiments were sourced from BD Biosciences, Beckman Coulter, and BioLegend. Information about the manufacturer name, catalogue number, and titrated volume of antibody utilised in the study is enlisted in Supplementary Table S3. The gating strategy utilised for the assay is available in Supplementary Figure S3.

### 4.3. Lymphocyte Subset Analysis (LSSA)

For LSSA, the PIDOT (Primary Immunodeficiency Orientation Tube) protocol recommended by the Euro Flow consortium was adapted [46,47,48,49]. A wash–stain–lyse–wash method was employed [46,47,48,49]. Briefly, 200 μL of whole blood (collected in an EDTA vacutainer) was taken in a FACS tube and washed/centrifuge with 3 mL of phosphate-buffered saline (PBS) at 250× *g* for 5 min (using a benchtop centrifuge and swing bucket rotor). After discarding the supernatant, the wash step was repeated. The cells were incubated for 20 min at room temperature in dark conditions with a pre-titrated 14-colour antibody cocktail comprising CD8 FITC (clone SK-1, BD Pharmigen, San Jose, CA, USA); IgD FITC (clone IA6-2, BioLegend, San-Diego, CA, USA); CD56 PE (clone N901, Beckman Coulter, Brea, CA, USA); CD16 PE (clone 3G8, BD Pharmigen, San Jose, CA, USA); CD27 PE-CF594 (clone MT271, BD Horizon, San Jose, CA, USA); CD4 PerCP Cy 5.5 (clone RPA-T4, BD Pharmigen, San Jose, CA, USA); IgM PerCP Cy 5.5 (clone MHM-88, BioLegend, San-Diego, CA, USA); CD19 PC7 (clone: J3-119, Beckman Coulter, Brea, CA, USA); TCRgd PC7 (clone IMMU-510, Beckman Coulter, Brea, CA, USA); CD3 APC (clone: UCHT1, Beckman Coulter, Brea, CA, USA); CD14 APC700 (clone: RMO52, Beckman Coulter, Brea, CA, USA); CD45 APCH7 (clone: 2D1, BD Pharmigen, San Jose, CA, USA); HLA-DR Pacific Blue (clone: IMMU-357, Beckman Coulter, Brea, CA, USA); and CD45RA BV510 (clone H100, BD Horizon, USA). After incubation, red blood cell (RBC) lysis was performed using 1 mL Optilyse C™ (Beckman coulter, San Jose, CA, USA) for 10 min at room temperature, and 2 mL of PBS was added for washing (250 g, 5 min). The supernatant was discarded, and the cells were resuspended in 250 μL of PBS. Data acquisition for all FCM experiments was carried out on either a DxFlex or Navios Ex flow cytometer (Beckman coulter, Brea, CA, USA) and analysed using Kaluza v2.1 software. Doublets, cell debris, and non-singlet populations were excluded using FSC versus SSC gating. The stopping gate was set at 100,000 total lymphocytes. The gating strategy of the assay is available in Supplementary Figure S3.

### 4.4. B Cell Immunophenotyping

For B cell immunophenotyping, the wash–stain–lyse–wash method was similarly employed. The antibodies used for surface staining included CD19 APC-Cy7 (clone SJ25C1, BD Pharmigen, San Jose, CA, USA); IgD FITC (clone IA6-2, BioLegend, San-Diego, CA, USA); IgM PerCP Cy 5.5 (clone MHM-88, BioLegend, San-Diego, CA, USA); CD24 PE (clone: ML5, BD Pharmigen, San Jose, CA, USA); CD27 PE-CF594 (clone MT271, BD Horizon, San Jose, CA, USA); CD10 APC 700 (clone: ALB1, Beckman Coulter, Brea, CA, USA); CD38 Pe-Cy-7 (clone: HIT2, BD Pharmigen, San Jose, CA, USA); and CD21 BV510 (clone: 1048, BD OptiBuild, San Jose, CA, USA). The stopping gate was set at 50,000 B cells. The gating strategy utilised for B cell immunophenotyping is available in Supplementary Figure S4.

### 4.5. T Regulatory Cell (Tregs) Evaluation

For Treg evaluation, a surface staining protocol was employed. An amount of 100 μL of whole-blood sample was taken in a FACS tube and was stained with an antibody cocktail containing CD3 ECD (clone: UCHT1, Beckman Coulter, USA); CD4 PE-Cy7 (clone:SK3, BD Pharmigen, USA); CD127 FITC (clone: HIL-7R-M21, BD Pharmigen, USA); and CD25 PE (clone: M-A251, BD Pharmigen, USA). The cells were incubated in the dark at room temperature for 20 min, followed by RBC lysis using 500 μL of Optilyse solution for 10 min. After lysis, the cells were washed with 2 mL of PBS, centrifuged at 250× *g* for 5 min, and resuspended in 250 μL of PBS. The stopping gate was set at 10,000 Tregs, identified as a CD3+ CD4+ CD25high CD127low population. The gating strategy utilised for the assay is available in Supplementary Figure S5.

### 4.6. Specific Protein Expression

(i) *CTLA4* expression

*CTLA4* protein expression was assessed using a whole-blood stimulation assay. For this assay, 200 μL of whole blood from heparinized vacutainers from both healthy controls and patients was added into FACS tubes with 2 conditions: unstimulated and stimulated conditions. To the stimulated tubes, 100 μL of PMA (Phorbol 12-Myristate 13-Acetate) (0.15 μg/mL) and Calcium Ionophore (CaI, 0.15 μg/mL) were added, while an equivalent volume of incomplete RPMI was added to the unstimulated tubes. An amount of 10 µL of CD152 APC (clone: BNI3, BD Pharmigen, USA) antibody was added in all tubes. The tubes were incubated at 37 °C in a CO_2_ incubator for 4 h. After incubation, the samples were washed with 1X PBS (250 g, 5 min, room temperature). The cell pellets were resuspended in an antibody cocktail [CD3 ECD (clone: UCHT1, Beckman Coulter, USA); CD4 PE-Cy7 (clone:SK3, BD Pharmigen, USA); CD127 FITC (clone: HIL-7R-M21, BD Pharmigen, USA); and CD25 PE (clone: M-A251, BD Pharmigen, USA)] and incubated in the dark for 20–30 min at room temperature. Following this, 2 mL of 1x BD FACS lysis solution was added and incubated for 5 min. The samples were centrifuged at 250× *g* for 5 min, washed, and analysed using a flow cytometer. *CTLA4* protein expression was measured on the CD3+CD4+CD25hiCD127low (Treg) population. For analysis, gating was established using the unstimulated condition, and the positive shift was quantified from the stimulated condition. Results were recorded as the percentage of shift, and interpretation was based on the positive shift observed in the healthy control, as shown in Supplementary Figure S6.

(ii) *LRBA* expression

*LRBA* protein expression was recently standardised by flow cytometry. Peripheral blood mononuclear cells (PBMCs) were isolated from 3 mL of sodium heparinized blood samples from patients and healthy controls using density gradient centrifugation. For stimulation, 1 × 10^6^ cells were plated in 96-well plates and stimulated with 100 μL PMA (Phorbol 12-Myristate 13-Acetate) (20 ng/mL) and CaI (Calcium Ionophore) (1 μg/mL) for 72 h at 37 °C in a CO_2_ incubator, while unstimulated cells were kept in media. After stimulation, cells were transferred to FACS tubes, washed with PBS (250 g, 5 min at room temperature), and stained with a surface antibody cocktail [CD3 APC (clone UCHT1, BD Pharmigen, USA); CD45 Krome Orange (clone J33, Beckman Coulter, USA); and CD69 FITC (clone: FN50, BD Pharmigen, USA)] for 30 min at room temperature in the dark. Fixation was performed using 70 μL of 10% formaldehyde in 1X PBS (freshly prepared) for strictly 10 min at room temperature, followed by permeabilization with 1 mL of 0.1% Triton X in PBS (prewarmed at 37 ˚C) for 30 min at 37 ˚C. The cells were washed with 1 mL of 0.05% Triton X prepared in BSA-PBS and incubated with rabbit polyclonal anti-*LRBA* antibody (in all tubes) for 1 h at room temperature in the dark. After washing with PBS, the cells were stained with a secondary F(ab’)_2_ donkey anti-rabbit IgG antibody–PE conjugate for 30 min (in all tubes) at room temperature in the dark. A final PBS wash was performed, and the cells were resuspended in 250 μL of PBS and acquired on a flow cytometer (Becton Dickinson, San Jose, CA, USA). *LRBA* protein expression was analysed on activated (CD69+) T cells, with results interpreted based on percentage positive shift as compared to the unstimulated condition, as shown in Supplementary Figure S7.

### 4.7. Molecular Analysis

Genomic DNA was extracted from peripheral blood with a Qiagen DNA extraction kit (Cat no’s:69504), according to the manufacture protocol. Targeted next-generation sequencing was performed on the majority of the patients by Medgenome Labs Pvt Ltd., India, or Life Cell Pvt Ltd., India. DNA was analysed using a targeted gene capture technique with a custom-designed capture kit. Libraries were sequenced to a mean depth of >80–100X using the Illumina sequencing platform. The genes covered in the panel are listed in Supplementary Table S4. Germline variants were identified following the GATK best practices framework, utilising Sentieon software. The sequencing reads were aligned to the human reference genome (GRCh38 or GRCh37) using the BWA aligner. Sentieon was employed for processing, including duplicate removal, base quality recalibration, and realignment of indels. Variant calling was conducted using the Sentieon haplotype caller, while detected germline variants underwent comprehensive annotation using the VariMAT pipeline. Gene-level annotation of variants was performed with the Variant Effect Predictor (VEP) tool against the Ensembl release 104 human gene model. In addition to identifying single-nucleotide variants (SNVs) and small insertions/deletions (indels), copy number variants (CNVs) were detected from targeted sequencing data using the ExomeDepth algorithm. Clinically significant mutations in coding and non-coding regions were annotated by referencing published literature and disease databases, including ClinVar, OMIM, HGMD, LOVD, DECIPHER (population CNVs), and SwissVar. Variants with high allele frequencies were filtered using data from population databases such as 1000 Genomes Project Phase 3, gnomAD (v3.1 and 2.1.1), dbSNP, GenomeAsia, and the Indian-specific MedVarDb (v4.0). The potential impact of non-synonymous variants was assessed using computational tools like PolyPhen-2, SIFT, MutationTaster2, and LRT. Clinically significant variants were prioritised for interpretation and were subsequently reported. Sanger sequencing was performed to validate the variants, followed by familial segregation depending on the availability of the parent’s sample.

Statistical analysis: Statistical analysis was performed on GraphPad Prism (version 9) software. To compare the two groups of patients, appropriate statistical tests including Mann–Whitney U, χ2, or Fisher’s exact tests were employed. All statistical tests were two-tailed, and a significance level of less than 0.05 was considered statistically significant.

## 5. Conclusions

Both CHAI and LATAIE have overlapping and variable clinical manifestations and immune abnormalities with only subtle differences between them. Recognising clinical and immunological clues in patients manifesting with recurrent infections, autoimmunity, and IBD is important for early diagnosis and appropriate management of these disorders.

## Figures and Tables

**Table 1:** Demographic details of patients with LATAIE and CHAI.

Parameters	LATAIE (n = 17)	CHAI (n = 12)	*p*-Value
# Sex ratio (M/F)	11/6	8/4	>0.999
# Consanguinity (%)	15 (88.24%)	0	<0.0001 ****
# Positive family history (%)	1 (5.88%)	7 (58.33%)	0.0033 **
## Median age of onset of symptoms (in years) (range)	4.5 (0.58–19)	13 (0.83–38)	0.0342 *
## Median age at diagnosis (in years) (range)	10 (0.75–38)	18 (5–55)	0.0108 *
## Median delay in diagnosis (in years) (range)	3 (0–28)	8.5 (1–42)	0.0917

# Fisher exact test was used. ##: Mann–Whitney test was employed. M = male, F = female; the values are represented as the median, and the range is represented as the maximum to minimum. Statistical significance is indicated by asterisks (*). **** represents a *p*-value < 0.0001, ** represents a *p*-value of 0.0033, and * indicates a *p*-value < 0.05. A *p*-value below 0.05 was considered statistically significant in this analysis, Patients with LATAIE predominantly presented symptoms early in life, with a median age of onset of 4.5 years (range: 0.58–19 years). In contrast, CHAI patients typically presented during adolescence, with a median age of onset of 13 years (range: 0.83–38 years) (*p*-value 0.0342). The median age at diagnosis was 10 years (0.75–38 years) for LATAIE and 18 years (5–55 years) for CHAI (*p*-value 0.0108). The median diagnostic delay was shorter for LATAIE patients at 3 years (0–28 years), compared to 8 years (1–42 years) in CHAI patients (*p*-value 0.0917).

**Table 2:** Summary of clinical manifestations observed in our cohort of LATAIE and CHAI.

Clinical Manifestations, n (%)	LATAIE (n = 17)	CHAI (n = 12)	*p*-Value
Autoimmune cytopenia, n (%)	12 (70.59)	9 (75.0)	>0.999
Splenomegaly, n (%)	10 (58.82)	1 (8.33)	0.0080 **
Lymphadenopathy, n (%)	7 (41.18)	6 (50.0)	0.7163
Sinopulmonary infections, n (%)	10 (58.82)	1 (8.33)	0.0080 **
Inflammatory bowel disease, n (%)	8 (47.06)	7 (58.33)	0.7104
Hepatomegaly, n (%)	6 (35.29)	1 (8.33)	0.1872
Chronic diarrhoea, n (%)	6 (35.29)	5 (41.67)	>0.999
Unexplained fevers, n (%)	5 (29.41)	3 (25)	>0.999
Other infections, n (%)	5 (29.41)	2 (16.67)	0.6645
Failure to thrive, n (%)	5 (29.41)	2 (16.67)	0.6645
Recurrent otitis media, n (%)	2 (11.76)	1 (8.33)	>0.999
Polyarthritis, n (%)	2 (11.76)	2 (16.67)	>0.999
Type 1 diabetes, n (%)	2 (11.76)	0	0.4975
Autoimmune hepatitis, n (%)	1 (5.88)	2 (16.67)	0.5534
Finger clubbing, n (%)	1 (5.88)	0	>0.999
Vasculitis, n (%)	1 (5.88)	0	>0.999
Interstitial lung disease, n (%)	1 (5.88)	0	>0.999
Aplastic anaemia, n (%)	0	1 (8.33)	0.4138

Fisher exact test was used to clinically compare the two entities. Statistically significant values are marked as asterisk **, where ** indicates degree of significance *p* < 0.05 is considered as significant

**Table 3:** Immune abnormalities in LATAIE and CHAI.

Immune Abnormalities	LATAIE	CHAI	*p*-Value
Lymphopenia (n = 20)	5 (35.71)	1(16.67)	0.6120
**Low Th naïve cell% (n = 20)**	**9 (64.29)**	**0**	0.0141 *
Low Tc naïve % (n = 20)	2 (14.29)	0	>0.9999
Elevated DNTs (n = 18)	6 (50)	2(33.33)	0.6380
Low Tregs % (n = 12)	4 (57.14)	0	0.0699
Low B cell % (n = 20)	9 (64.29)	2 (33.33)	0.3359
Low B memory (n = 20)	6 (42.86)	3 (50)	>0.999
Low class switch memory B cells (n = 20)	3 (21.43)	2 (33.33)	0.6126
Hypogammaglobulinemia (n = 21)	6 (42.86)	1(14.29)	0.3371
**Only IgG low (n = 21)**	**1(7.14)**	**4 (57.14)**	0.0251 *

The parameters marked in bold are statistically significant between the two categories, *p* < 0.05. Statistical significance is indicated by asterisks (*), and * indicates degree of significance. Th naïve: CD4+CD45RA+CD27+; Tc naïve: CD8+ CD45RA+CD27+; DNTs: Double-negative T cells characterised as CD3+CD4-CD8-TCRab+ cells (>2.5% of total T cells); Tregs: T regulatory cells characterised as CD3+CD4+ CD25 high CD127low (normal range: 3–9% of CD4+T cells, unpublished in-house range). B memory: CD19+CD27+; Class switch memory B cells: CD19+CD27+IgM-IgD−; CD21 low B cells: CD19+CD21low+CD38−. immunoglobulin (Ig) level values were compared to the reference ranges available for all these populations. Data for this is provided in Supplementary Table S1a,b.

**Table 4:** Molecular spectrum of LATAIE and CHAI cohort.

Patient	Variant Description					Variant Classification According to ACMG Guidelines	Reported/Novel	Treatment	Outcome
	Location	cDNA Position	Protein Change	Zygosity	Domain Affected				
L1	Intron 29	c.4729+1G>C	5′splice site	Homo	-	PVS1, PM2 (likely Pathogenic I)	Novel	Managed with steroids (6 months, poor response) and MMF (initial improvement for AICs and lymphoproliferation, later developed infections).	Expired due to pneumonia and ARDS
L2	Exon 8	c.999del	p.Phe333LeufsTer29	Homo	ConA/LAMG #	PVS1, PM2, PP4 (Pathogenic Ic)	Novel	Managed with platelet transfusions for Evans syndrome and MMF * for 1 year (poor response). Currently on methylprednisolone (for 5 months).	Alive
L3	Exon 26	c.4333C>T	p.Arg1445Ter	Homo	DUF 4707	PVS1, PM2, PM3, PP5 (Pathogenic Ib)	[25]	-	Lost to follow-up
L4	Exon 25	c.4086_4087del	p.Gln1363SerfsTer25	Homo	between DUF4704 and WD40	PVS1, PM2 (Likely Pathogenic I)	Novel	Treated with intravenous (IV) antibiotics for 4 months due to infection complications.	Lost to follow-up
L5	Exon 23	c.3662_3663insGA	p.T1222Kfs*4	Homo	between DUF4704 and WD40	PVS1, PM2 (Likely Pathogenic I)	Novel	Required multiple transfusions and pulse steroids for hemolysis but showed poor control. Later developed IBD/AIHA and was started on methotrexate with IVIG for 8 months. Remains refractory to most therapies.	Alive and has been offered a transplant
L6	Exon 28	c.4522C>T	p.Gln1508Ter	Homo	between WD1 and DUF1088	PVS1, PM2, PM3, PP4 (Pathogenic Ib)	[26]	Receiving monthly IVIG for ITP for 1.5 years, with good response.	Alive
L7	Exon 21	c.2479C>T	p.Arg827Ter	Homo	between DUF4704 and WD40	PVS1, PM2, PM3, PP5 (Pathogenic Ib)	[27]	-	Lost to follow-up
L8	Exon 53	c.7799G>A	p.C2600Y	Homo	WD40 (WD2)	BS2, BP6 (Likely Benign-I)	[18]	-	Lost to follow-up
L9	Exon 2	c.217-1G>A	3′splice site	Homo	Near N-terminal region	PVS1,PM2 (Likely Pathogenic-I)	Novel	Receiving monthly IVIG for hypogammaglobulinemia for 2 years.	Alive
L10	Exon 50	c.7523_7524del	p.P2508Rfs*23	Homo	BEACH	PVS1, PM2 (Likely Pathogenic-I)	[19]	Partial response to sirolimus for autoimmunity.	Alive
L11	Exon 20	c.2449C>T	p.Gln817Ter	Homo	between DUF4704 and WD40	PVS1, PM2, PM3, PP5 (Pathogenic Ib)	[19]	Started on Rituximab for hepatosplenomegaly and positive family of EBV lymphoproliferation. Developed Evans syndrome months later, managed with corticosteroids. A year later, had CMV reactivation and was treated with oral valganciclovir. Currently has complete control of symptoms and lymphoproliferation on sirolimus and IVIG.	Alive
L12	Exon 30	c.4759_4762delACTA	p.Thr1587ArgfsTer28	Homo	between WD1 and DUF1088	PVS1, PM2, PM3, PP5 (Pathogenic Ib)	[19]	Insulin for 10 months for T1D. Refractory Evans at 2 years, treated with methylprednisolone, rituximab, azathioprine, vincristine, and IVIG for hypogammaglobulinemia.	Transplant offered but declined. Died from pneumonia and ARDS.
L13	Exon 30; Exon 26	c.4759_4762delACTA; c.4300_4301delAT	p.Thr1587ArgfsTer28; p.Met1434ValfsTer33	Comp Het	between WD1 and DUF1088	PVS1, PM2, PM3, PP5 (Pathogenic Ib) PVS1, PM2 (Likely Pathogenic-I)	[19,28]	Sirolimus for AIHA, partial response. Abatacept initiated with poor response.	Underwent successful HSCT. Alive
L14	Exon 20	c.2449C>T	p.Gln817Ter	Homo	between DUF4704 and WD40	PVS1, PM2, PM3, PP5 (Pathogenic Ib)	[19]	Lost to follow-up.	-
L15	Intron 11	c.1494-2A>G	3′ splice site	Homo	-	PVS1, PM2 (Likely Pathogenic-I)	Novel	Corticosteroids for autoimmune manifestations and insulin for T1D.	Alive
L16	Intron 25	c. 4159-1 G>T	3′ splice site	Homo	-	PVS1, PM2, PM3, PP5 (Pathogenic Ib)	Novel	Treated with rituximab, IVIG, and sirolimus for autoimmune manifestations.	Alive
L17	Exon 25; Exon 55	c.4124T>G; c.8128T>C	p.Ile1375Arg; p.Cys2710Arg	Comp Het	between DUF4704 and WD40; WD4	PM2, PP3 (VUS); PM2, PP3 (VUS)	Novel	Steroid, IVIG, methotrexate, cotrimoxazole.	Alive
C1	Exon 2	c.223C>T	p.Arg75Trp	Het	LBD *	PS1, PS4, PP1, PM2, PM1, PP2, PP3 (Pathogenic II)	[29]	Monthly IVIG for hypogammaglobulinemia for 1 year.	Alive
C2	Exon 2	c.346delA	p.Ile116SerfsTer4	Het	LBD *	PVS1, PM2, PP4 (Pathogenic Ic)	Novel	Transfusion-dependent, post-diagnosis was started on abatacept, responded.	Alive
C3	Exon 2	c.221T>C	p.Leu74Pro	Het	LBD *	PM2, PM1, PP2 (Likely Pathogenic V)	Novel	IV antibiotics. Developed Hodgkin’s, on chemotherapy.	Alive
C4	Exon 2	c.401T>G	p.Met134Arg	Het	LBD *	PM2, PM1, PP2 (Likely Pathogenic V)	[13]	AIHA and IBD phenotype managed with low-dose Sirolimus and steroid.	Alive
C5	Exon 2	c.436G>A	p.Gly146Arg	Het	LBD *	PS1, PS2, PS4, PM2, PM1, PP2 (Pathogenic II)	[30]	Lost to follow-up.	-
C6	Exon 2	c.221T>C	p.Leu74Pro	Het	LBD *	PM2, PM1, PP2, PP4 (Likely Pathogenic V)	Novel	Cotrimoxazole for pneumonia and diarrhoea.	Alive
C7	Exon 2	c.416A>G	p.Tyr139Cys	Het	LBD *	PS4, PM2, PM1, PM5, PP2 (Pathogenic IIIa)	[31]	-	-
C8	Exon 2	c.416A>G	p.Tyr139Cys	Het	LBD *	PS4, PM2, PM1, PM5, PP2 (Pathogenic IIIa)	[31]	-	-
C9	Exon 2	c.416A>G	p.Tyr139Cys	Het	LBD *	PS4, PM2, PM1, PM5, PP2 (Pathogenic IIIa)	[31]	-	-
C10	Exon 2	c.151 C>T	p.Arg51Ter	Het	LBD *	PS4, PVS1, PM2 (Pathogenic Ia)	[32]	Received multiple therapies for colitis, autoimmune manifestations, steroids, sirolimus, and azathioprine.	Alive
C11	Exon 2	c.151 C>T	p.Arg51Ter	Het	LBD *	PS4, PVS1, PM2 (Pathogenic Ia)	[32]	IVIG *** for ITP.	Alive
C12	Exon 3	c.529del	p.Tyr177IlefsTer10	Het	TmD **	PVS1, PM2 (Likely Pathogenic I)	Novel	Platelet transfusion-dependent, managed with eltrambopag and menabol for pancytopenia.	Expired due to unknown reasons

Homo: homozygous; CompHet: compound heterozygous. * MMF: mycophenolate mofetil: # ConA/LAMG: concanavalin A-like lectin/glucanase domain superfamily. ND: not done. * LBD: ligand-binding domain. ** TmD: transmembrane domain.*** IVIG: intravenous immunoglobulin therapy.

## Data Availability

All data generated or analysed during this study are included in this published article [and its Supplementary Information files]. The processed sequence data supporting this study are available at ClinVar under accession numbers: SCV00540247, SCV005402469, SCV005627726, SCV005402747, SCV005402748, SCV005627727, SCV005627739, SCV005627740, SCV005402749, SCV005627744, SCV005627745, SCV005627746, SCV005627747, SCV005402750, SCV005402751, SCV005402752, SCV005407740, SCV005627748, SCV005407741, SCV005407742, SCV005627749, SCV005627750, SCV005627751, SCV005627752, and SCV005407743.

## Supplementary Materials

The following supporting information can be downloaded at https://www.mdpi.com/article/10.3390/ijms27010014/s1.

## Abbreviations

The following abbreviations are used in this manuscript:

AIHA	Autoimmune haemolytic anaemia
APC	Allophycocyanin
APCs	Antigen-presenting cells
APC700	Allophycocyanin-700
APCH7 or APC-750	Allophycocyanin-750
BEACH	Beige and Chediak–Higashi
BV421	Brilliant violet 421
BV510	Brilliant violet 510
CaI	Calcium Ionophore
CD	Cluster of differentiation
CHAI	*CTLA4* haploinsufficiency with autoimmune infiltration
CTLA-4	Cytotoxic T lymphocyte antigen-4
CVID	Common variable immunodeficiency
DNA	Deoxyribose nucleic acid
DUF1088	Domain of unknown function 1088
ECD	Electron-coupled dye
FITC	Fluorescein isothiocyanate
HSCT	Hematopoietic stem cell transplantation
IBD	Inflammatory bowel disease
IEI	Inborn errors of immunity
ILD	Interstitial lung disease
ITP	Immune thrombocytopenia
IVIG	Intravenous immunoglobulin
LATAIE	*LRBA* deficiency with autoantibodies, regulatory T (Treg) cell defects, autoimmune infiltration, and enteropathy
*LRBA*	Lipopolysaccharide-responsive beige-like anchor protein
MMF	mycophenolate mofetil
PB	Pacific Blue
PE-Cy7 or PC7	Phycoerythrin-cyanine7 conjugate
PE	Phycoerythrin
PeCF594	PE and CF594, tandem conjugate dye
PH	Pleckstrin homology
PIDOT	Primary Immunodeficiency Orientation Tube
PIRDs	Primary immune regulatory disorders
PMA	Phorbol 12-Myristate 13-Acetate
RPMI 1640	Roswell Park Memorial Institute 1640
TCR	T cell receptor
Tregs	T regulatory cells
VUS	Variant of Uncertain significance
