# Supplementary material for: Clinical, Genetic, and Immunological Spectrum of CHAI and LATAIE Patients from a Tertiary Referral Centre in India
Source: Int J Mol Sci. 2025 Dec 19;27(1):14. doi: 10.3390/ijms27010014 (PMC12785805; doi:10.3390/ijms27010014)
Supplement: Supplementary file 1 [file ijms-27-00014-s001.zip › ijms-3760285_R2Supplementary Figures.pptx]

## Slide 1
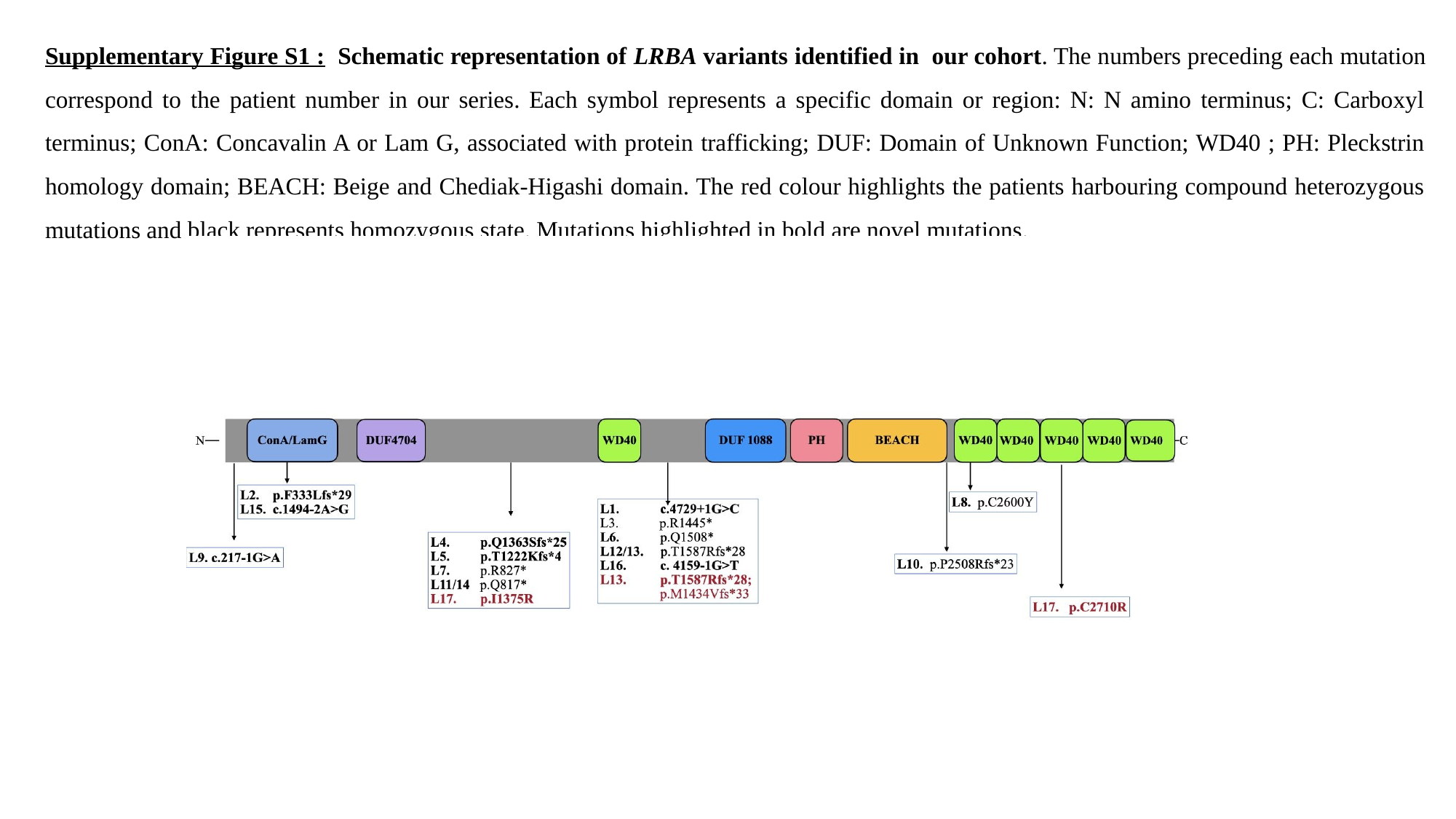

Supplementary Figure S1 : Schematic representation of LRBA variants identified in our cohort. The numbers preceding each mutation correspond to the patient number in our series. Each symbol represents a specific domain or region: N: N amino terminus; C: Carboxyl terminus; ConA: Concavalin A or Lam G, associated with protein trafficking; DUF: Domain of Unknown Function; WD40 ; PH: Pleckstrin homology domain; BEACH: Beige and Chediak-Higashi domain. The red colour highlights the patients harbouring compound heterozygous mutations and black represents homozygous state. Mutations highlighted in bold are novel mutations.

## Slide 2
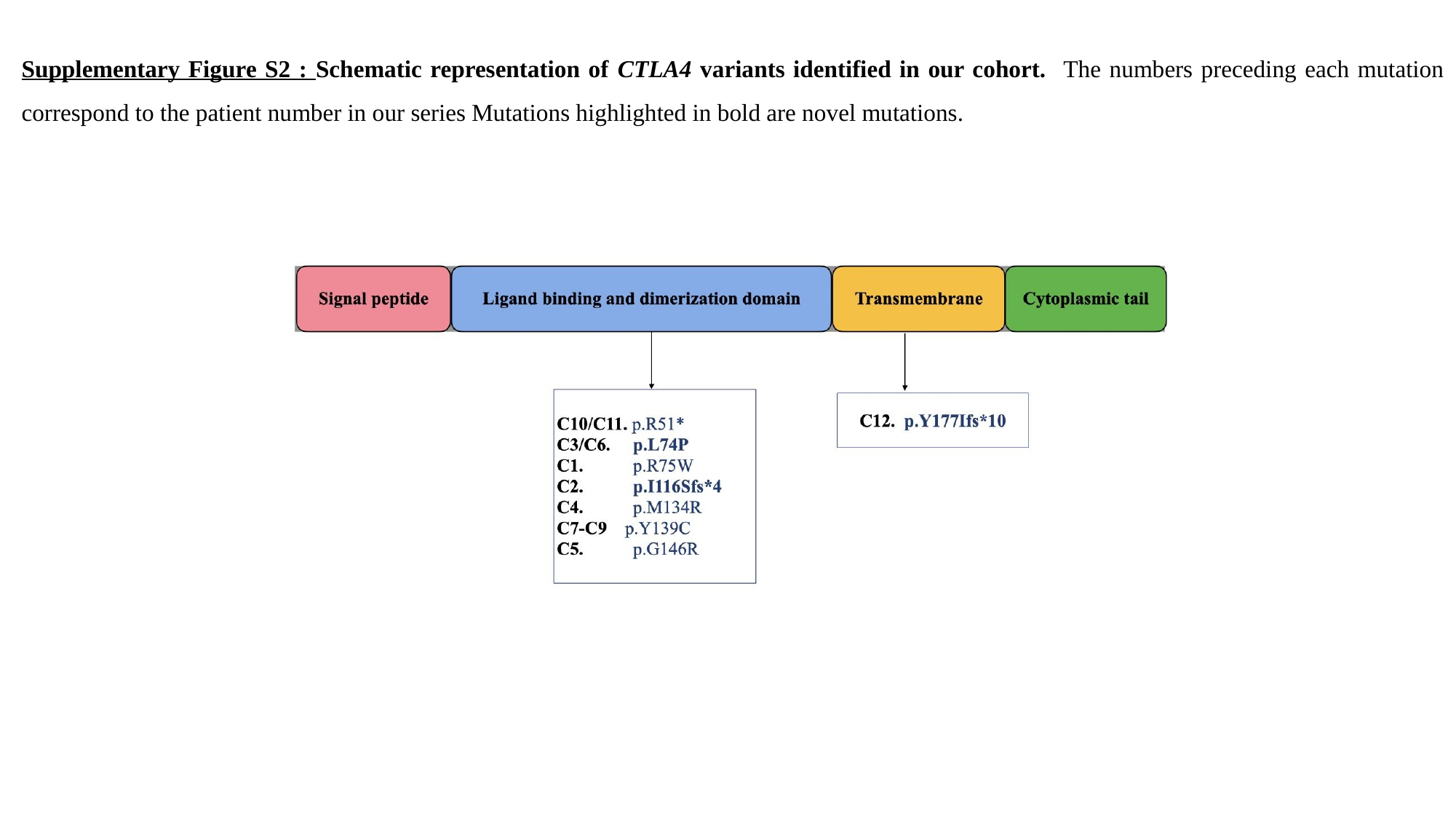

Supplementary Figure S2 : Schematic representation of CTLA4 variants identified in our cohort. The numbers preceding each mutation correspond to the patient number in our series Mutations highlighted in bold are novel mutations.

## Slide 3
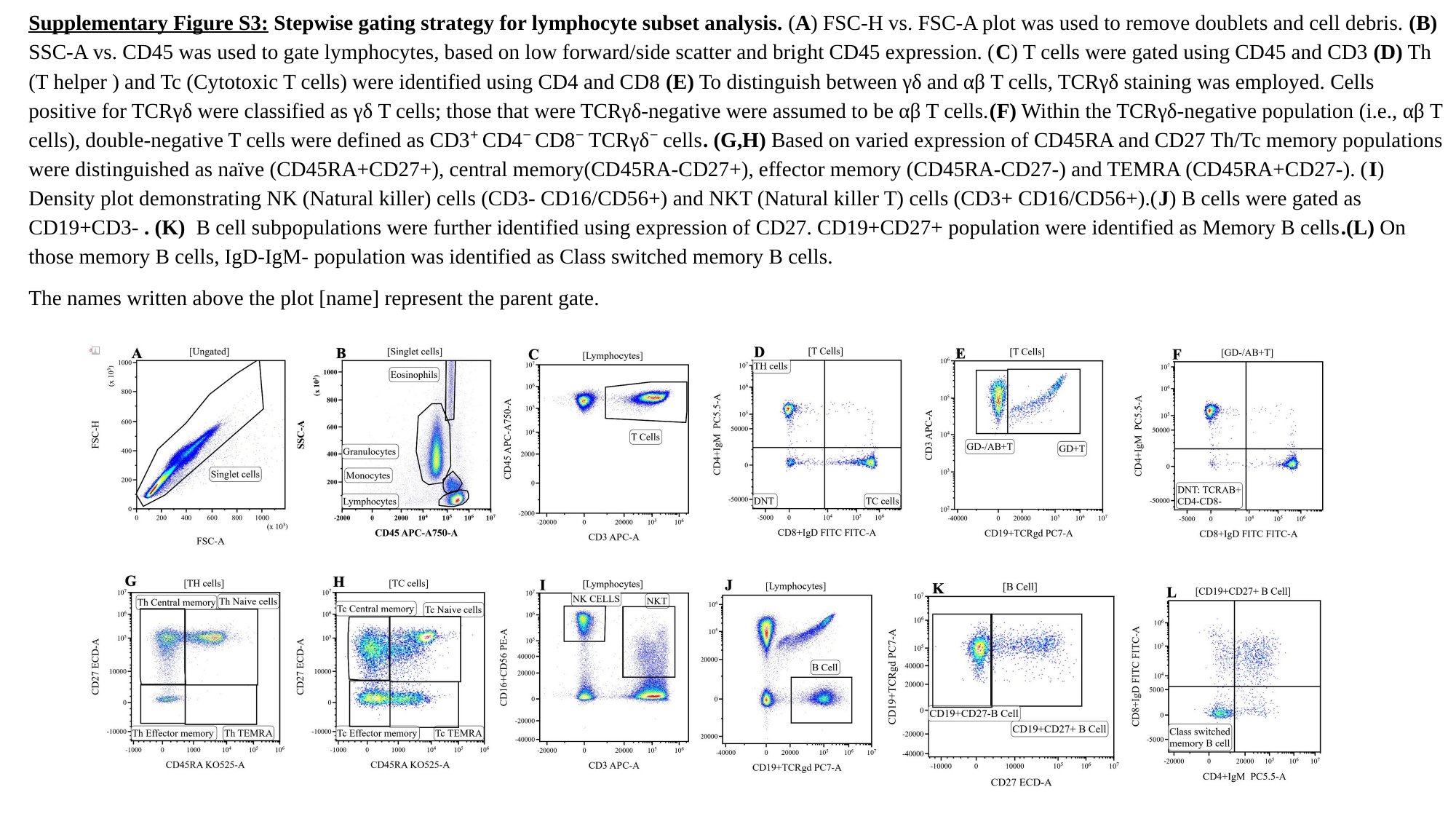

Supplementary Figure S3: Stepwise gating strategy for lymphocyte subset analysis. (A) FSC-H vs. FSC-A plot was used to remove doublets and cell debris. (B) SSC-A vs. CD45 was used to gate lymphocytes, based on low forward/side scatter and bright CD45 expression. (C) T cells were gated using CD45 and CD3 (D) Th (T helper ) and Tc (Cytotoxic T cells) were identified using CD4 and CD8 (E) To distinguish between γδ and αβ T cells, TCRγδ staining was employed. Cells positive for TCRγδ were classified as γδ T cells; those that were TCRγδ-negative were assumed to be αβ T cells.(F) Within the TCRγδ-negative population (i.e., αβ T cells), double-negative T cells were defined as CD3⁺ CD4⁻ CD8⁻ TCRγδ⁻ cells. (G,H) Based on varied expression of CD45RA and CD27 Th/Tc memory populations were distinguished as naïve (CD45RA+CD27+), central memory(CD45RA-CD27+), effector memory (CD45RA-CD27-) and TEMRA (CD45RA+CD27-). (I) Density plot demonstrating NK (Natural killer) cells (CD3- CD16/CD56+) and NKT (Natural killer T) cells (CD3+ CD16/CD56+).(J) B cells were gated as CD19+CD3- . (K) B cell subpopulations were further identified using expression of CD27. CD19+CD27+ population were identified as Memory B cells.(L) On those memory B cells, IgD-IgM- population was identified as Class switched memory B cells.
The names written above the plot [name] represent the parent gate.

## Slide 4
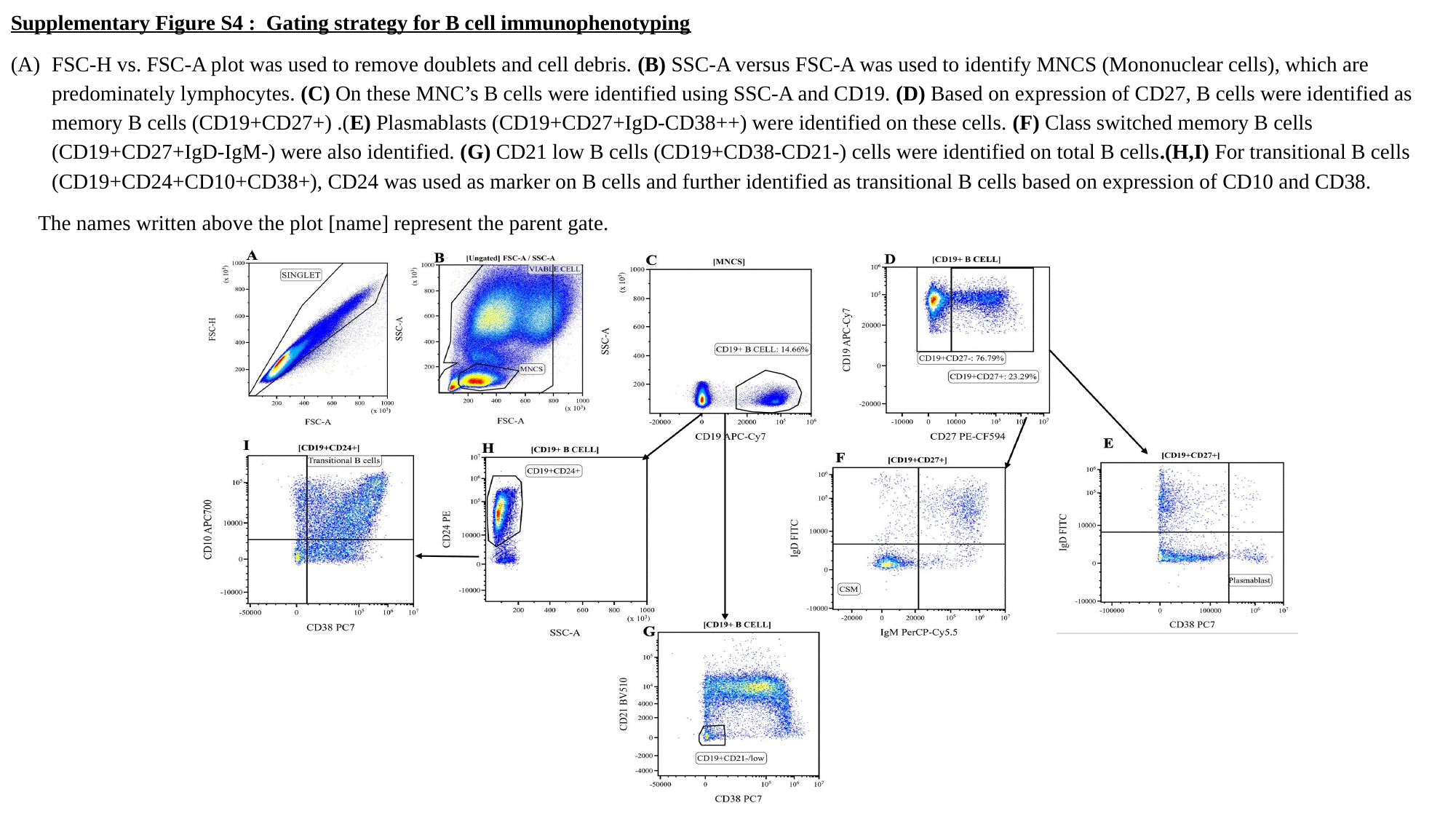

Supplementary Figure S4 : Gating strategy for B cell immunophenotyping
FSC-H vs. FSC-A plot was used to remove doublets and cell debris. (B) SSC-A versus FSC-A was used to identify MNCS (Mononuclear cells), which are predominately lymphocytes. (C) On these MNC’s B cells were identified using SSC-A and CD19. (D) Based on expression of CD27, B cells were identified as memory B cells (CD19+CD27+) .(E) Plasmablasts (CD19+CD27+IgD-CD38++) were identified on these cells. (F) Class switched memory B cells (CD19+CD27+IgD-IgM-) were also identified. (G) CD21 low B cells (CD19+CD38-CD21-) cells were identified on total B cells.(H,I) For transitional B cells (CD19+CD24+CD10+CD38+), CD24 was used as marker on B cells and further identified as transitional B cells based on expression of CD10 and CD38.
The names written above the plot [name] represent the parent gate.

## Slide 5
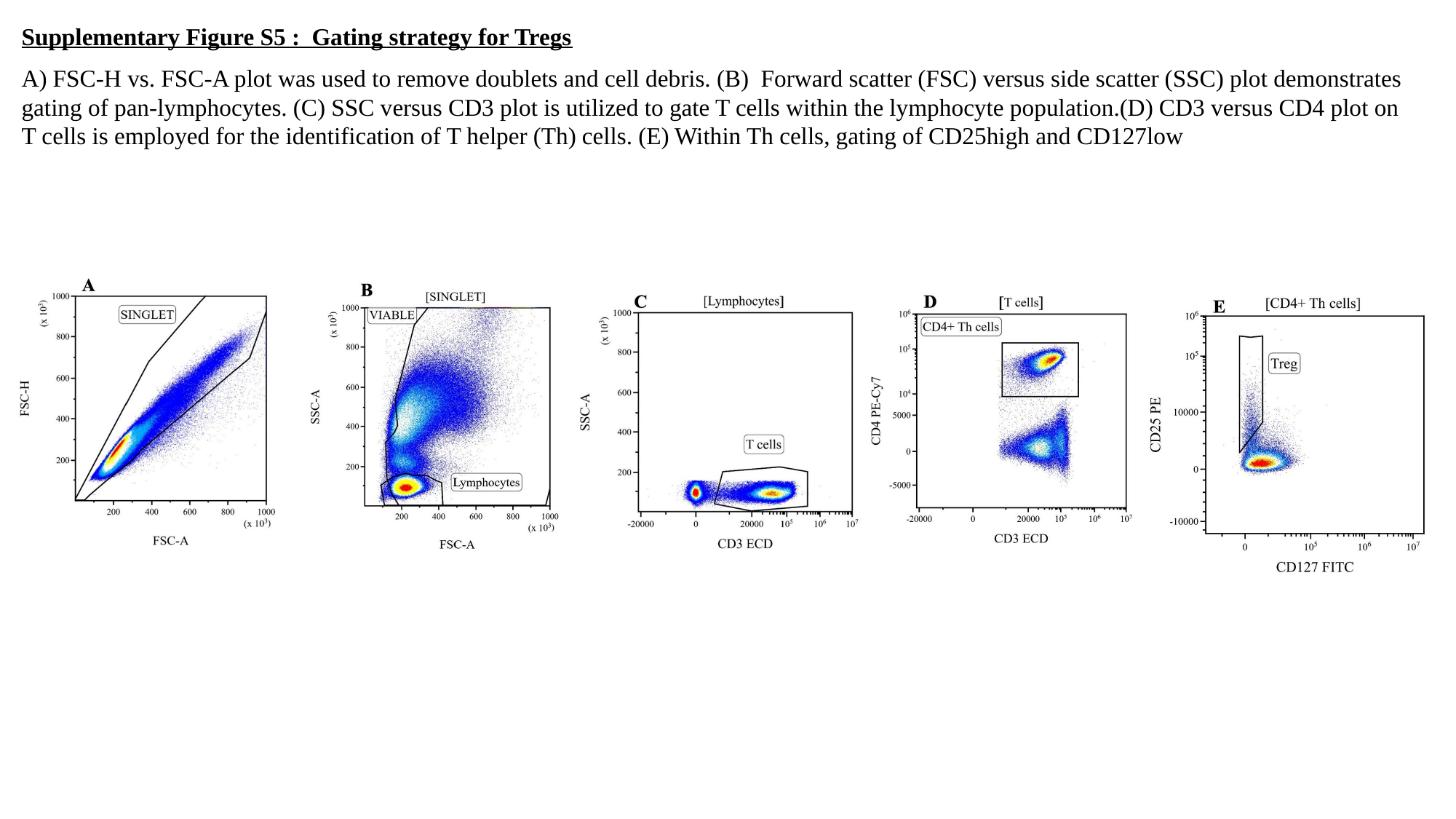

Supplementary Figure S5 : Gating strategy for Tregs
A) FSC-H vs. FSC-A plot was used to remove doublets and cell debris. (B) Forward scatter (FSC) versus side scatter (SSC) plot demonstrates gating of pan-lymphocytes. (C) SSC versus CD3 plot is utilized to gate T cells within the lymphocyte population.(D) CD3 versus CD4 plot on T cells is employed for the identification of T helper (Th) cells. (E) Within Th cells, gating of CD25high and CD127low

## Slide 6
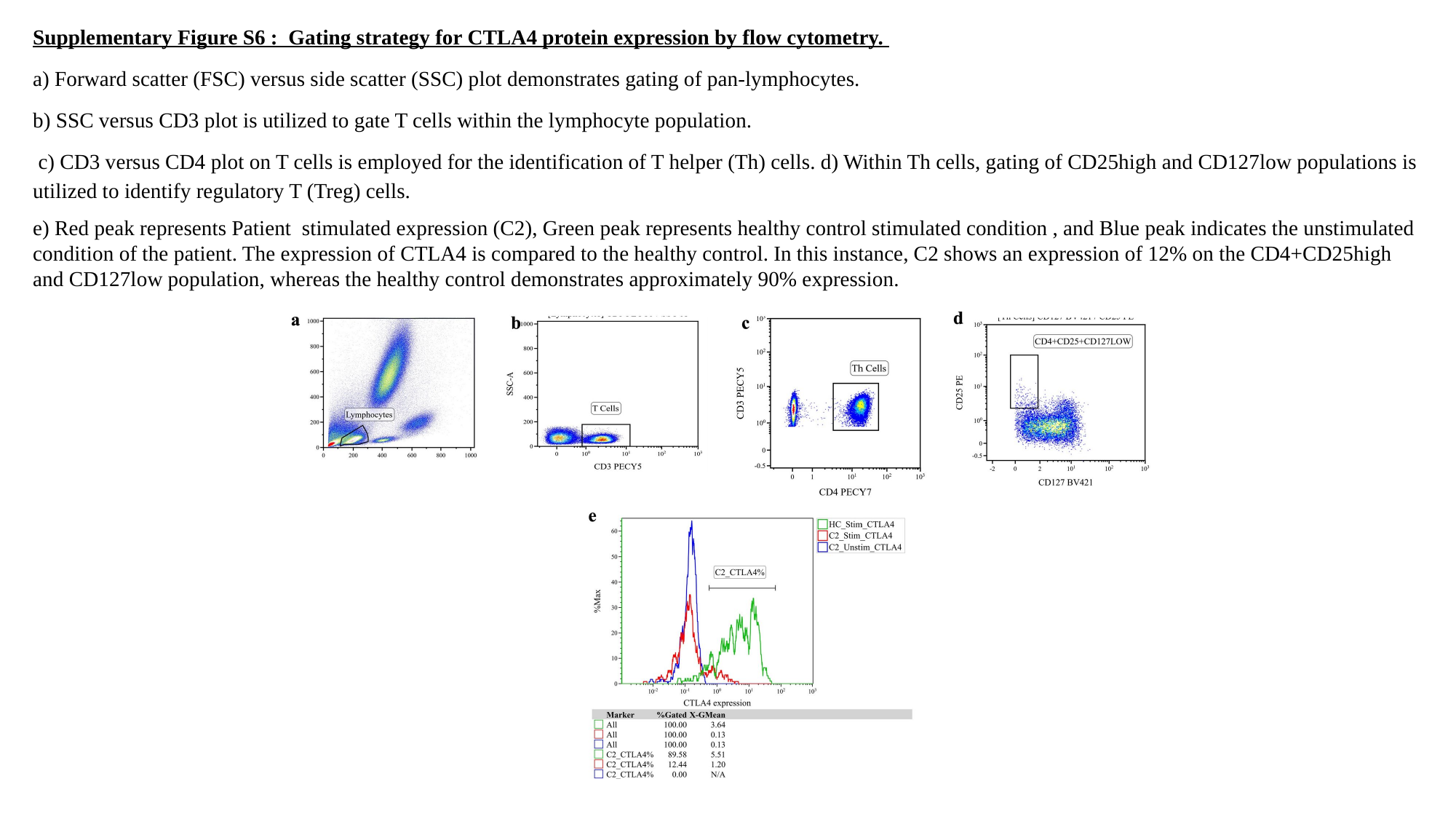

Supplementary Figure S6 : Gating strategy for CTLA4 protein expression by flow cytometry.
a) Forward scatter (FSC) versus side scatter (SSC) plot demonstrates gating of pan-lymphocytes.
b) SSC versus CD3 plot is utilized to gate T cells within the lymphocyte population.
 c) CD3 versus CD4 plot on T cells is employed for the identification of T helper (Th) cells. d) Within Th cells, gating of CD25high and CD127low populations is utilized to identify regulatory T (Treg) cells.
e) Red peak represents Patient stimulated expression (C2), Green peak represents healthy control stimulated condition , and Blue peak indicates the unstimulated condition of the patient. The expression of CTLA4 is compared to the healthy control. In this instance, C2 shows an expression of 12% on the CD4+CD25high and CD127low population, whereas the healthy control demonstrates approximately 90% expression.

## Slide 7
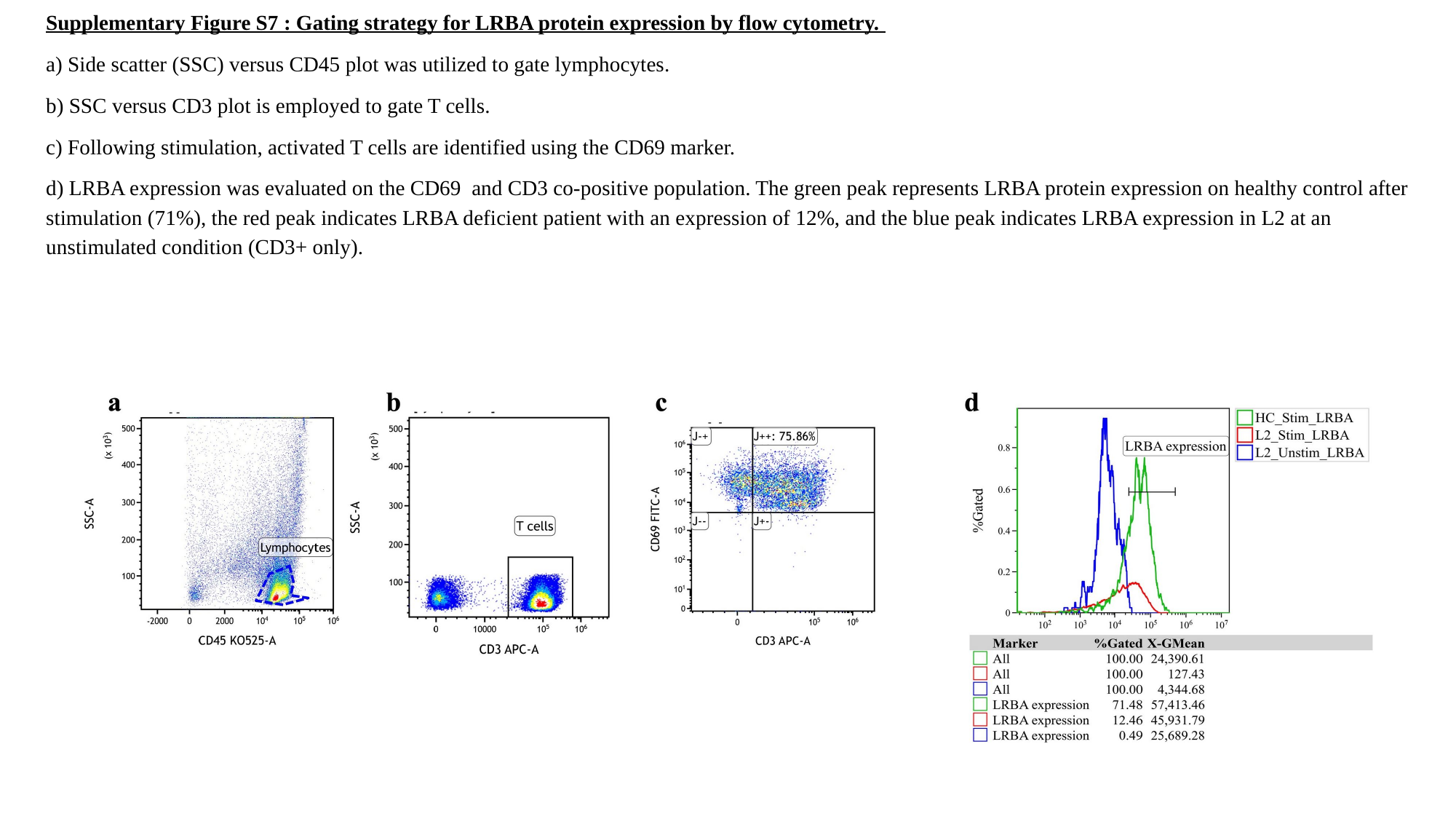

Supplementary Figure S7 : Gating strategy for LRBA protein expression by flow cytometry.
a) Side scatter (SSC) versus CD45 plot was utilized to gate lymphocytes.
b) SSC versus CD3 plot is employed to gate T cells.
c) Following stimulation, activated T cells are identified using the CD69 marker.
d) LRBA expression was evaluated on the CD69 and CD3 co-positive population. The green peak represents LRBA protein expression on healthy control after stimulation (71%), the red peak indicates LRBA deficient patient with an expression of 12%, and the blue peak indicates LRBA expression in L2 at an unstimulated condition (CD3+ only).
