# Supplementary material for: Clinical, Genetic, and Immunological Spectrum of CHAI and LATAIE Patients from a Tertiary Referral Centre in India
Source: Int J Mol Sci. 2025 Dec 19;27(1):14. doi: 10.3390/ijms27010014 (PMC12785805; doi:10.3390/ijms27010014)
Supplement: Supplementary file 1 [file ijms-27-00014-s001.zip › ijms-3760285_R2_Supplementary Table.pdf]

Supplementary Table S1 : Immunological findings in patients with LATAIE

Patient	L1	L2	L3	L4	L5	L6	L7	L8	L9	L10	L11	L12	L13	L14
WBC count (counts/mm3)	1200	4920	3380	10610	7500	9910	1700	18230	6890	2760	10250	16410	7300	8880
Hb (gm/dl)	7.9	8.3	6.9	11.5	8.9	10.9	11.1	6.7	11.5	11.6	12.6	9.2	8.8	15.8
Platelet counts (/microlitre)	161	5	2	287	150	47	3	279	378	9	334	18	56	186
Neutrophils %	2.4	87.6	44	58.5	-	32	46	58	67	53	54	63	45.3	70
Lymphocytes %	72	9.6	40	24.2	-	49	38	35	24	36	34.4	28.8	43.6	23
Absolute lymphocyte count (ALC)	893	940	1352	2568	1838	4806	649	6381	1654	994	3560	4759	3212	2078
Normal range	2300-5400	1400-3300	2300-5400	1900-3700	1400-3300	3400-9000	1900-3700	2300-5400	1400-3300	1900-3700	1900-3700	2300-5400	1900-3700	1400-3300
B cell %	30	10	16	2	5	17	9	28	3	7	14	8	8	1
Absolute B cell count	268	94	216	51	92	816	58	1787	50	70	488	381	257	21
Normal range	390-1400	110-570	390-1400	270-860	110-570	610-2600	270-860	390-1400	110-570	270-860	270-860	390-1400	270-860	110-570
T cell %	64	82	76	86	94	76	87	69	90	90	71	85	88	69
Absolute T cell count	571	771	1028	2208	1728	3653	565	4403	1489	894	2474	4045	2827	1435
Normal range	1400-3700	1000-2200	1400-3700	1200-2600	1000-2200	1900-5900	1200-2600	1400-3700	1000-2200	1200-2600	1200-2600	1400-3700	1200-2600	1000-2200
Th cell %	29	59	42	21	66	36	29	46	24	45	34	43	42	27
Absolute Th cell count	259	555	568	539	1213	1730	253	2935	397	447	1185	2046	1349	556
Normal range	700-2200	530-1300	700-2200	650-1500	530-1300	1400-4300	650-1500	700-2200	530-1300	650-1500	650-1500	700-2200	650-1500	530-1300
Tc cell %	28	19	26	59	23	34	41	20	60	40	32	39	41	38
Absolute Tc cell	250	179	352	1515	423	1634	266	1276	992	397	1115	1856	1317	801
Normal range	490- 1300	330-920	490-1300	370-1100	330-920	500-1700	370-1100	490-1300	330-920	370-1100	370-1100	490-1300	370-1100	330-920
NK cell %	5	8	3	10	2	5	3	1	6	2	12	5	3	27
Absolute NK cell count	45	75	41	257	37	248	19	64	99	20	418	238	96	561
Normal range	130-720	70-480	130-720	100-480	70-480	160-920	100-480	130-720	70-480	100-480	100-480	130-720	100-480	70-480
IgG levels(g/L)	10.8	6.8	2.01	1.4	3.3	12.8	3.91	4.3	3.6	7.85	2.04	3.5	14.9	3.31
Normal range	3.5-16.2	6.4-17	3.5-16.2	6.5-16.2	6.4-17	3.5-16.2	6.5-16.2	4.5-15.9	6.4-17	4-15.9	3.5-16.2	3.5-16.2	4-15.9	6.6-16.9
IgA levels(g/L)	1.1	1.07	0.02	0.278	0.369	1.07	0.29	0.933	0.35	0.536	<0.239	<0.239	0.797	<0.279

Normal range	0.17-3.18	0.52- 4.68	0.17-3.18	0.57-5.43	0.52-4.68	0.01-0.91	0.57-5.43	0.17-2.9	0.52-4.68	0.17-2.90	0.17-3.18	0.17-3.18	0.17-2.90	0.57-5.43
IgM levels(g/L)	0.841	0.82	**0.23**	**5.62**	0.44	**1.94**	**0.23**	0.778	0.15	0.92	**0.168**	**0.168**	1.19	**0.192**
Normal range	0.30-2.65	0.34-3.48	0.30-2.65	0.30-2.65	0.34-3.48	0.30-1.83	0.30-2.65	0.34-3.48	0.34-3.48	0.24-3.48	0.30-2.65	0.30-2.65	0.34-3.48	0.37-2.58
IgE levels (IU/ml)	128	17.8	21	**1.72**	53	48.3	18.4	432	8	20.9	17.1	6.4	42	17.8
Normal range	3-423	3-423	3-423	3-423	3-423	3-423	3-423	3-423	3-423	3-423	3-423	3-423	3-423	3-423
Th naïve %	**20**	**10**	**18**	**19**	**13**	56	**31**	58	44	52	**36**	32.8	**35**	**7**
Normal range	52-92	16-100	52-92	37-97	16-100	56-100	37-97	52-92	37-97	46-99	46-99	46-99	46-99	16-100
Tc naïve %	43	38	44	**7**	36	49	51	68	**16**	43	42	37	28	12
Normal range	19-100	6-100	19-100	20-95	6-100	10-100	20-95	19-100	20-95	16-100	16-100	16-100	16-100	6-100
Tregs (3-9% of Th cells)	-	4.6	-	-	**2.2**	**2.89**	-	-	-	-	4.8	10	**1.5**	**0.79**
B memory	10	14	**4.5**	**0.82**	**3.1**	5.3	12	30	**2.2**	18	**2.8**	**7.5**	6.2	15
Normal range	8.1-33.3	7-29	9-24.3	9-35	7-29	3.5-12.2	9-35	8.1-33.3	7-29	8.1-33.3	8.1-33.3	8.1-33.3	8.1-33.3	7-29
Class switch memory B cells	5	6.4	1.7	**0.1**	0.3	1.93	3.9	14	**0.08**	11	**0.3**	3	2.78	1.6
Normal range	0.8-10.3	0.2-11.7	0.7-6.3	1.3-8.8	0.2-11.7	0.3-1.7	1.3-8.8	0.8-10.3	1.2-9.4	1.1-14	1.1-14	0.8-10.3	0.8-10.3	0.2-11.7
CD21 low B cells (1.1-6.9%)	-	5.3	-	**26**	**11**	**40**	**20**	-	**7.9**	-	-	-	-	-
Transitional B cells(0.6-3.5%)	-	2	-	3	**0.22**	**0.2**	**5.5**	-	2	-	-	-	-	-
Plasma blasts (0.4-3.6%)	-	1.9	-	**6**	**0.3**	1.8	**0.3**	-	**0.09**	-	-	-	-	-
DNT % (<2.5% of T cells)	**7.87**	**2.9**	**6.76**	1.83	1.92	2.45	**4.5**	-	0.5	-	**4.15**	1.93	**2.67**	2.3
Protein expression by flow cytometry^	ND	**12.46%**	ND	ND	ND	**10%**	ND	ND	ND	ND	ND	ND	ND	ND

^Normal range for LRBA protein expression is more than 70% . Immunological workup was not done in L15, L16, and L17.

Bold values marked with red represent those below the normal range, while bold values marked with green indicate values above the normal range

Supplementary Table S2 : **Immunological findings in CHAI patients**

Patient	C1	C2	C3	C4	C5	C6	C7
WBC count (counts/mm3)	**16.3**	9.1	3.72	8.26	**3.92**	5.75	-
Hb(gm/dl)	**8.1**	**10.3**	17	**9.8**	14	11.2	-
Platelet counts(/ microlitre)	553	298	371	995	15	451	-
Neutrophils %	51	62	55	52	**26**	54	-
Lymphocytes %	37	29	**21.5**	28	45	34	-
Absolute lymphocyte count (ALC) (range)	6031	2369	**818**	2288	1764	1932	-
Normal range	3400-9000	1400-3300	1400-3300	1400-3300	1400-3300	1400-3300	-
B cell %	**34**	**6**	10	**5**	**19**	**18**	-
Absolute B cell count	2053	158	**82**	114	335	348	-
Normal range	610-2600	110-570	110-570	110-570	110-570	110-570	-
T cell %	60	85	71	**91**	76	78	-
Absolute T cell count	3622	**2243**	**581**	2082	1341	1507	-
Normal range	1900-5900	1000-2200	1000-2200	1000-2200	1000-2200	1000-2200	-
Th cell %	39	33	34	56	34	37	-
Absolute Th cell count	2354	871	**278**	1281	600	715	-
Normal range	1400-4300	530-1300	530-1300	530-1300	530-1300	530-1300	-
Tc cell %	18	**48**	34	33	31	38	-
Absolute Tc cell	1087	**1287**	**278**	755	547	734	-
Normal range	500-1700	330-920	330-920	330-920	330-920	330-920	-
NK cell %	6	8	17	4	**3**	**2.6**	-

Absolute NK cell count	362	211	139	92	**53**	**58**	-
Normal range	160-950	70-480	70-480	70-480	70-480	70-480	-
IgG levels	**5.96**	**3.8**	**4.83**	**6.4**	8.78	**17.3**	**0.653**
Normal range	6.5-16.2	6.5-16.2	6.6-16.4	6.4-17	6.6-16.4	6.5-16.2	6.5-16.2
IgA levels	8.24	1.63	**0.38**	0.82	0.341	2.81	1.49
Normal range	0.57-5.43	0.57-5.43	0.57-5.43	0.52-4.68	0.57-5.43	0.52-4.68	0.57-5.43
IgM levels	4.68	0.7	**0.36**	0.83	0.337	1.48	0.41
Normal range	0.30-2.65	0.30-2.65	0.37-2.58	0.34-3.48	0.30-2.65	0.39-3.38	0.30-2.65
IgE levels (IU/ml)	-	<17.2	17.2	17.8		96.5	10
Normal range	3-423	3-423	3-423	3-423	3-423	3-423	3-423
Th naïve %	45	53	18	-	42	34	-
Normal range	16-100	37-97	16-100	37-97	37-97	16-100	
Tc naïve %	41	67	31	85	40.74	27	-
Normal range	6-100	20-95	6-100	20-95	20-95	6-100	
Tregs (3-9% of Th cells)	4.3	**13**	3.5	3.7	-	3.2	-
B memory	**2.5**	**2.8**	**2.42**	6.8	11.88	19	-
Normal range	7-29	7-29	7-29	7-29	7-29	7-29	
Class switch memory B cells	1.4	**0**	0.6	**1**	3.94	8	-
Normal range	0.2-11.7	1.2-9.4	0.2-11.7	1.2-9.4	0.2-11.7	1.2-9.4	-
CD21 low B cells (1.1-6.9%)	**48**	**-**	**-**	**56**	**-**	**29**	-
Transitional B cells 0.6-3.5%)	0.88	-	-	1	-	**11**	-
Plasma blasts(0.4-3.6%)	2.5	-	-	1.4	-	0.6	-
DNT % (<2.5% of T cells)	1.52	**2.6**	2	1.1	**3.58**	0.7	-
Protein expression by flow cytometry^	**14%**	**12%**	ND	**20%, low MFI**	ND	**26%**	ND

^: Normal range for healthy controls for protein expression is >80%.Immunological workup was not available for C8-C12. Bold values marked with red represent those below the normal range, while bold values marked with green indicate values above the normal range

Supplementary Table S3: **Antibodies used for screening using flow cytometry.**

Antigen/ Reagents	Fluorochrome (clone)	Manufacturer, country of origin	Catalogue	Titrated volume/ test
**Lymphocyte subset analysis**				
CD8	FITC (SK-1)	BD Pharmigen, USA	555634	5 µl
IgD	FITC (IA6-2)	BioLegend, USA	348205	0.6 µl
CD56	PE (N901)	Beckman Coulter , USA	A07788	2.5µl
CD16	PE (3G8)	BD Pharmigen, USA	555407	5 µl
CD27	Pe-CF594 (MT271)	BD Horizon, USA	562297	0.3 µl
CD4	PerCP Cy5.5 (RPA-T4)	BD Pharmigen, USA	560650	2 µl
IgM	PerCP Cy5.5 (MHM-88)	BioLegend, USA	314511	0.6 µl
CD19	PC7 (J3-119)	Beckman Coulter , USA	IM3628U	1 µl
TCRgd	PC7 (IMMU-510)	Beckman Coulter, USA	B10247	0.5 µl
CD3	APC (UCHT1)	Beckman Coulter, USA	IM2467U	2 µl
CD14	APC700 (RMO52)	Beckman Coulter, USA	A99020	1 µl

CD45	APCH7 (2D1)	BD Pharmigen, USA	560178	0.5 µl
HLA-DR	Pacific Blue (IMMU-357)	Beckman Coulter, USA	B36291	2.5 µl
CD45RA	BV510 (H100)	BD Horizon USA	563031	0.3 µl
OptiLyse C	-	Beckman Coulter, USA	A11895	1 ml
**T-Regulatory cell panel**				
CD3	ECD (UCHT1)	Beckman Coulter, USA	A07748	2 µl
CD4	PE-CY7 (SK3)	BD Pharmigen, USA	557852	0.5 µl
CD127	FITC (HIL-7R-M21)	BD Pharmigen, USA	560549	2.5 µl
CD25	PE (M-A251)	BD Pharmigen, USA	555432	5 µl
**B cell panel**				
CD19	APC-Cy7 (SJ25C1)	BD Pharmigen, USA	557791	1 µl
IgD	FITC (IA6-2)	BioLegend, USA	348205	0.6 µl
IgM	PerCP Cy5.5 (MHM-88)	BioLegend, USA	314511	0.6 µl
CD24	PE (ML5)	BD Pharmigen, USA	555428	2.5 µl
CD27	Pe-CF594 (MT271)	BD Horizon, USA	562297	0.3 µl

CD10	APC 700 (ALB1)	Beckman Coulter, USA	A86358	1 µl
CD21	BV510 (1048)	BD OptiBuild, USA	742760	0.5 µl
CD38	Pe-CY7 (HIT2)	BD Pharmigen, USA	560677	1 µl
**LRBA protein expression**				
Rabbit polyclonal Anti- LRBA antibody	Purified	Sigma	HPA023597	2 µl
Secondary F(ab)2 donkey anti rabbit IgG antibody	PE	BD Phosflow, USA	558416	5 µl
CD45	Krome Orange (J33)	Beckman Coulter, USA	A96416	5 µl
CD3	APC (HIT3a)	BD Pharmigen, USA	555342	2 µl
CD69	FITC (FN50)	BD Pharmigen, USA	555530	5 µl
Phorbol 12-Myristate 13- Acetate (PMA)	-	Sigma	P-8139	-
Calcium Ionophore (CaI)	-	Sigma	I-0634	-
Incomplete RPMI1640 Medium	-	GIBCO ADVANCED RPMI 1640(1X); L-Glutamine	12633-012	-

BD FACS Lysing solution 10X concentrate	-	BD Biosciences, USA	349202	-
Preparation of 1X lysing solution : 1ml of BD FACS Lysing solution 10X concentrate +9ml of distilled water				
Paraformaldehyde solution	-	Sigma	47608	-
10 % formaldehyde prepared in 1X PBS (Freshly prepared only before use) (Fixative)				
Bovine Serum albumin (lyophilized powder)	-	Sigma	SRE0096	-
Triton X	-	Sigma	9036-19-5	-
**Permeabilization buffer**: 0.1 % Triton X in 1X PBS (Prewarmed at 37 ˚C)<br>**Wash buffer:** 0.05% Triton X in 1% BSA- 1X PBS				
**CTLA4 protein expression panel**				
CD3	ECD (UCHT1)	Beckman Coulter, USA	A07748	2 µl
CD3	PE-Cy 5 (HIT3a)	BD Pharmigen, USA	561006	2.5 µl
CD4	PE-CY7 (SK3)	BD Pharmigen, USA	557852	0.5 µl
CD127	FITC (HIL-7R-M21)	BD Pharmigen, USA	560549	2.5 µl
CD25	PE (M-A251)	BD Pharmigen, USA	555432	5 µl

CTLA4 (CD152)	APC (BNI3)	BD Pharmigen, USA	555855	10 µl

Supplementary Table S4 : Genes included in the NGS panel were sequenced with mean coverage of 80-100X .

Genes included in the NGS panel					
ACP5	*ACTB*	*ADA*	*CLPB*	*COPA*	*CORO1A*
ADAM17	*ADAR*	*AICDA*	*CR2*	*CSF2RA*	*CSF2RB*
AIRE	*AK2*	*AP1S3*	*CSF3R*	*CTC1*	*CTLA4*
AP3B1	*AP3D1*	*APOL1*	*CTPS1*	*CTSC*	*CXCR4*
ATM	*ATP6AP1*	*B2M*	*CYBA*	*CYBB*	*DCLRE1C*
BCL10	*BCL11B*	*BLM*	*DDX58*	*DKC1*	*DNAJC21*
BLNK	*BTK*	*C1QA*	*DNMT3B*	*DOCK2*	*DOCK8*
C1QB	*C1QC*	*C1R*	*ELANE*	*EPG5*	*ERCC6L2*
C1S	*C2*	*C3*	*EXTL3*	*FAAP24*	*FADD*
C5	*C6*	*C7*	*FAS*	*FASLG*	*FAT4*
C8A	*C8B*	*C8G*	*FCGR3A*	*FCN3*	*FERMT3*
C9	*CARD11*	*CARD14*	*CLPB*	*COPA*	*CORO1A*
CARD9	*CASP10*	*CASP8*	*CR2*	*CSF2RA*	*CSF2RB*
CCBE1	*CD19*	*CD247*	*CSF3R*	*CTC1*	*CTLA4*
CD27	*CD3D*	*CD3E*	*CTPS1*	*CTSC*	*CXCR4*
CD3G	*CD40*	*CD40LG*	*CYBA*	*CYBB*	*DCLRE1C*
CD46	*CD55*	*CD59*	*DDX58*	*DKC1*	*DNAJC21*
CD79A	*CD79B*	*CD81*	*DNMT3B*	*DOCK2*	*DOCK8*
CD8A	*CDCA7*	*CEBPE*	*ELANE*	*EPG5*	*ERCC6L2*
CECR1	*CFB*	*CFD*	*EXTL3*	*FAAP24*	*FADD*

CFH	*CFHR1*	*CFHR2*	*FAS*	*FASLG*	*FAT4*
CFHR3	*CFHR4*	*CFHR5*	*FCGR3A*	*FCN3*	*FERMT3*
CFI	*CFP*	*CFTR*	*FOXN1*	*FOXP3*	*FRP1*
CHD7	*CIITA*	*CLCN7*	*G6PC3*	*G6PD*	*GATA2*
GFI1	*HMOX1*	*IFNAR2*	*IGHM*	*IKBKB*	
IL10RA	*HAX1*	*HELLS*	*NOP10*	*OBFC1*	*ORAI1*
IL12RB1	*ICOS*	*IFIH1*	*OSTM1*	*OTULIN*	*PARN*
IL17RC	*IFNGR1*	*IFNGR2*	*PEPD*	*PGM3*	*PIK3CD*
IL2RA	*IGKC*	*IGLL1*	*PIK3R1*	*PLCG2*	*PLEKHM1*
IL7R	*IKZF1*	*IL10*	*PMS2*	*PNP*	*POLA1*
IRAK4	*IL10RB*	*IL12B*	*POLE*	*POLE2*	*PRF1*
IRF7	*IL17F*	*IL17RA*	*PRKCD*	*PRKDC*	*PSEN1*
ITCH	*IL1RN*	*IL21R*	*PSENEN*	*PSMB8*	*PSTPIP1*
JAGN1	*IL2RG*	*IL36RN*	*PTEN*	*PTPRC*	*RAB27A*
KDM6A	*INO80*	*IRAK1*	*RAC2*	*RAG1*	*RAG2*
LAT	*IRF2BP2*	*IRF3*	*RANBP2*	*RASGRP1*	*RBCK1*
LIG4	*IRF8*	*ISG15*	*RFX5*	*RFXANK*	*RFXAP*
LIG4	*ITGB2*	*ITK*	*RHOH*	*RLTPR*	*RMRP*
LYST	*JAK1*	*JAK3*	*RNASEH2A*	*RNASEH2B*	*RNASEH2C*
MAP3K14	*KMT2D*	*LAMTOR2*	*RNF168*	*RNF31*	*RNU4ATAC*
MEFV	*LCK*	*LIG1*	*RORC*	*RPSA*	*RTEL1*
MS4A1	*LPIN2*	*LRBA*	*SAMD9*	*SAMD9L*	*SAMHD1*
MTHFD1	*MAGT1*	*MALT1*	*SBDS*	*SEMA3E*	*SERPING1*
MYSM1	*MASP2*	*MCM4*	*SH2D1A*	*SH3BP2*	*SLC29A3*
NCF2	*MKL1*	*MOGS*	*SLC35C1*	*SLC37A4*	*SLC46A1*

NDNL2	*MSH6*	*MSN*	*SMARCAL1*	*SMARCD2*	*SNX10*
NFKB2	*MVK*	*MYD88*	*SP110*	*SPINK5*	*STAT1*
NHP2	*NBAS*	*NBN*	*STAT2*	*STAT3*	*STAT5B*
NLRP12	*NCF4*	*NCSTN*	*STIM1*	*STK4*	*STX11*
TFRC	*TRAF3IP2*	*THBD*	*STXBP2*	*TAP1*	*TAP2*
TINF2	*TTC37*	*TIRAP*	*TAPBP*	*TAZ*	*TBK1*
TMC6	*UNC13D*	*TMC8*	*TBX1*	*TCF3*	*TCIRG1*
TNFAIP3	*USB1*	*TNFRSF11A*	*TCN2*	*TERC*	*TERT*
TNFRSF13C	*VPS45*	*TNFRSF1A*	*TICAM1*	*TNFSF11*	*TPP2*
TNFSF12	*TLR3*	*TRAC*	*TMEM173*	*TREX1*	*TNFRSF13B*
TTC7A	*TNFRSF4*	*UNC93B1*	*TPP1*	*USP18*	*TRAF3*
WAS	*TRNT1*	*WRAP53*	*TYK2*	*WIPF1*	
